# Radiolytic Hydrogen Production in the Subseafloor Basaltic Aquifer

**DOI:** 10.3389/fmicb.2016.00076

**Published:** 2016-02-04

**Authors:** Mary E. Dzaugis, Arthur J. Spivack, Ann G. Dunlea, Richard W. Murray, Steven D’Hondt

**Affiliations:** ^1^Graduate School of Oceanography, University of Rhode Island, NarragansettRI, USA; ^2^Department of Earth and Environment, Boston University, BostonMA, USA

**Keywords:** radiolysis, hydrogen, basalt, ocean crust, geochemistry, deep biosphere

## Abstract

Hydrogen (H_2_) is produced in geological settings by dissociation of water due to radiation from radioactive decay of naturally occurring uranium (^238^U, ^235^U), thorium (^232^Th) and potassium (^40^K). To quantify the potential significance of radiolytic H_2_ as an electron donor for microbes within the South Pacific subseafloor basaltic aquifer, we use radionuclide concentrations of 43 basalt samples from IODP Expedition 329 to calculate radiolytic H_2_ production rates in basement fractures. The samples are from three sites with very different basement ages and a wide range of alteration types. U, Th, and K concentrations vary by up to an order of magnitude from sample to sample at each site. Comparison of our samples to each other and to the results of previous studies of unaltered East Pacific Rise basalt suggests that significant variations in radionuclide concentrations are due to differences in initial (unaltered basalt) concentrations (which can vary between eruptive events) and post-emplacement alteration. However, there is no clear relationship between alteration type and calculated radiolytic yields. Local maxima in U, Th, and K produce hotspots of H_2_ production, causing calculated radiolytic rates to differ by up to a factor of 80 from sample to sample. Fracture width also greatly influences H_2_ production, where microfractures are hotspots for radiolytic H_2_ production. For example, H_2_ production rates normalized to water volume are 190 times higher in 1 μm wide fractures than in fractures that are 10 cm wide. To assess the importance of water radiolysis for microbial communities in subseafloor basaltic aquifers, we compare electron transfer rates from radiolysis to rates from iron oxidation in subseafloor basalt. Radiolysis appears likely to be a more important electron donor source than iron oxidation in old (>10 Ma) basement basalt. Radiolytic H_2_ production in the volume of water adjacent to a square cm of the most radioactive SPG basalt may support as many as 1500 cells.

## Introduction

The oceanic basement contains the largest aquifer on Earth. Its fractured rock contains nearly 2% of Earth’s total volume of seawater (Johnson and Pruis, 2003). Although the extent of life and microbial activity in oceanic basement is not well known, a variety of evidence suggests that microbes reside within the aquifer (Cowen et al., 2003; Edwards et al., 2012; Jungbluth et al., 2013; Lever et al., 2013; Orcutt et al., 2013). Fisk et al. (1998) and Staudigel et al. (2008) report weathering textures suggestive of microbial alteration in subseafloor basaltic glass. Microorganisms have been found in fluid flowing through 3.5 million year old basalt on the Juan de Fuca ridge flank (Cowen et al., 2003). DNA and isotopic signatures of mineral alteration provide evidence of microbes and microbial activity in ridge-flank basalt (Lever et al., 2013).

Physical and chemical properties limit microbial habitability of the oceanic basement. For example, habitability within subseafloor basalt is constrained by availability of electron donors [e.g., organic carbon, ferrous iron (Fe^2+^), and hydrogen (H_2_)] and electron acceptors [e.g., oxygen (O_2_), nitrate (NO_3_^-^), and sulfate (SO_4_^2-^)] (Madigan et al., 2000; Bach and Edwards, 2003; D’Hondt et al., 2004). A number of studies have suggested that microbial life in igneous-rock aquifers may be supported by oxidation of electron donors native to the rock or produced by water–rock interactions (Pedersen, 1993; Stevens and McKinley, 1995; Kelley et al., 2001; Chapelle et al., 2002; Bach and Edwards, 2003; Edwards et al., 2005). For example, basement basalt has high concentrations of reduced elements, specifically iron (Fe) and sulfur (S) (Bach and Edwards, 2003). Oxidation of these elements with O_2_ or NO_3_^-^ in the seawater provides energy that microorganisms might utilize. Water–rock interactions that can produce electron donors in the form of molecular H_2_ include serpentinization (Kelley et al., 2001) and radiolysis of water due to radioactive decay of radionuclides within the rock (Pedersen, 1993; Lin et al., 2005a,b; Blair et al., 2007; D’Hondt et al., 2009, 2015; Edwards et al., 2011; Lollar et al., 2014). Water radiolysis within subseafloor basalt is the focus of this study.

Water radiolysis is the decomposition of water molecules by ionizing radiation produced during the decay of radioactive elements (Debierne, 1914; Le Caër, 2011). The principal radioactive elements that produce ionizing radiation in basalt are uranium (^238^U and ^235^U), thorium (^232^Th), and potassium (^40^K), which collectively emit alpha (α), beta (β), and gamma (γ) radiation as they and their daughter nuclides decay. Transfer of energy from this radiation excites and ionizes water molecules, producing several chemical species: e_aq_^-^, HO•, H•, HO_2_•, H_3_O^+^, OH^-^, H_2_O_2_, and H_2_ (Spinks and Woods, 1990; Le Caër, 2011). The distribution and rate of formation of these products depends on the linear energy transfer (LET) of the radiation (the amount of energy deposited by the radiation along its path). Low-LET radiation (γ-rays and β particles) ionizes water discretely along the radiation path. High-LET radiation (e.g., α particles) deposits energy densely along the particles’ track. The radiolytic yields of the radicals decline with LET. The radicals are short-lived and highly reactive; they recombine in the radiation track to produce stable decomposition products (H_2_ and H_2_O_2_). Production of H_2_ and H_2_O_2_ increases with increasing LET (Pastina and LaVerne, 2001; Le Caër, 2011).

For subseafloor environments, we are particularly interested in the production of the reductant H_2_. Many organisms catabolically utilize H_2_, including methanogens, sulfate-reducers, iron reducers, and nitrate reducers (Fang and Zhang, 2011). There is evidence that some of these organisms, specifically sulfate reducers and methanogens, are active in subseafloor basalt (Lever et al., 2013). Radiolysis undoubtedly occurs in subseafloor basalt, as both water and radiation are present. Edwards et al. (2012) suggested that in the old and relatively weathered basaltic basement of the South Pacific Gyre (SPG), radiolytic H_2_ may be the dominant electron donor.

The potential of radiolytic H_2_ as an electron donor has been studied for other environments, such as continental crust (Pedersen, 1993; Lin et al., 2005a,b) and deep-sea sediment (Blair et al., 2007). In these environments, water radiolysis supplies H_2_ that may support microbial communities. In deep-sea sediment, H_2_ may be especially important to microbial communities where organic carbon availability is very low (Blair et al., 2007; D’Hondt et al., 2009).

Here, we focus on calculating the rate of radiolytic H_2_ production in oceanic basement. More specifically, we use our recently published water radiolysis model to calculate H_2_ production rates in fractures of South Pacific subseafloor basalt (Dzaugis et al., 2015). Our model more accurately quantifies radiolytic H_2_ production in fractured hard rock then previous models, which are more appropriate for physically homogenous environments where solid particles are small compared to the distance that the ionizing radiation travels, such as deep-sea sediment.

To determine variations in radionuclide concentrations of relatively old SPG basalt, we analyzed basalt samples from three SPG sites with basement ages of circa 13 to 100 Ma. Our samples include a wide variety of alteration types. To identify effects of basement age, initial composition, and alteration on radiolysis rates, we compare our H_2_ production calculations for these samples to calculations for which we use previously published U, Th, and K data from young basalt samples on the East Pacific Rise (EPR) (Gale et al., 2013). In addition, we quantify the effect of fracture width on the H_2_ production rates. We assess the possible significance of radiolytic H_2_ production for microbial communities in subseafloor basaltic aquifers by comparing the electron transfer flux from radiolytic H_2_ to the electron transfer flux from iron oxidation in the basalt. Finally, we estimate the number of cells that might be supported by radiolysis in the basement.

## Materials and Methods

### Materials

**FIGURE 1:**
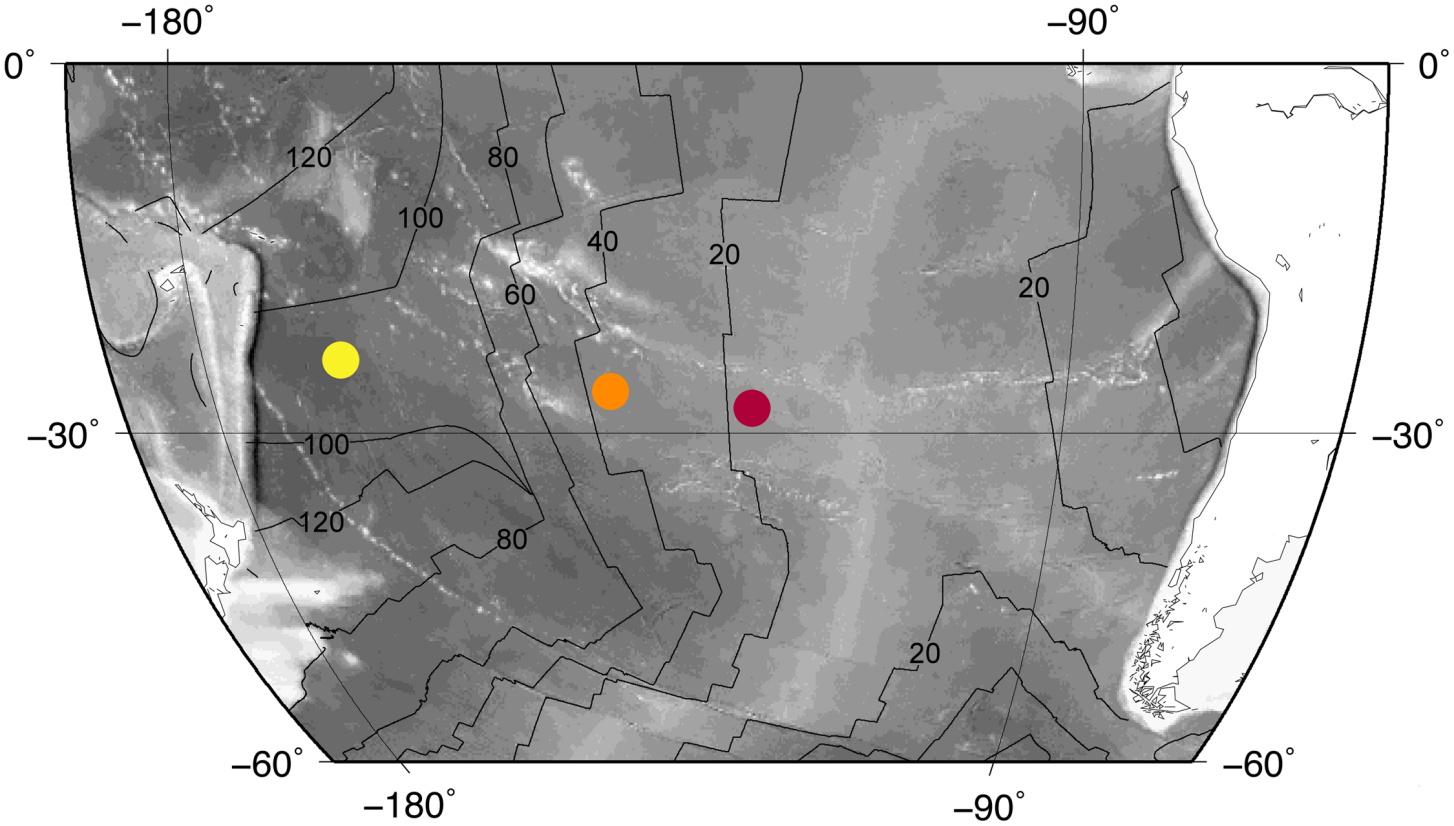
**Site locations.** Colored dots mark site locations: Site U1365 (yellow), Site U1367 (orange), Site U1368 (red). The site localities are superimposed on a bathymetry map with the East Pacific Rise in light gray. Black lines indicate basement age (20 Myr contours).

Integrated Ocean Drilling Program (IODP) Expedition 329 collected subseafloor basalt samples at three sites (Sites U1365, U1367, U1368; **Figure 1**). We analyzed 43 samples from these sites to determine the range of U, Th, and K concentrations in SPG basalt.

Site U1365 is located in the western part of the SPG, where the seafloor is approximately 5695 m below sea level (Expedition 329 Scientists, 2011a). At this location, basement is mainly composed of massive lava flows that are approximately 100 Ma (Dunlea et al., 2015a). Recovery of cored basalt was 74.6% at this site. Throughout the cored sequence, alteration extent of the recovered basalt varies between 2 and 95%, with alteration mainly associated with veins and vesicles near flow margins and breccias (Expedition 329 Scientists, 2011a). We analyzed 17 samples from Site U1365 for this study.

The second site, U1367, is east of Site U1365, with seafloor approximately 4288 m below sea level (Expedition 329 Scientists, 2011b). The basement is younger at this site, with an approximate age of 33.5 Ma. The uppermost basement is mostly composed of fractured pillow fragments with a small thin flow at the base of the recovered basalt. Due to the fractured nature of the basalt cored at this site, core recovery was low (11.2%). The extent of alteration in recovered basalt from this site varies between 2 and 25%. Most basalt alteration at Site U1367 occurs around vein-filled fractures of chilled margins (Expedition 329 Scientists, 2011b). We analyzed five samples from Site U1367.

Site U1368 is the eastern-most site of Expedition 329. Seafloor here is 3740 m below sea level (Expedition 329 Scientists, 2011c). Basement at Site U1368 is approximately 13.5 Ma. Core recovery was 27.6%. The recovered portion of the basement is dominantly composed of pillow basalt. Basalt alteration at this site is mainly in veins, vesicles, along chilled margins, and within volcaniclastic breccias. The recovered basalt varies in alteration extent from 2 to 60% (Expedition 329 Scientists, 2011c). There are 21 samples from Site U1368 used in this study.

### Alteration Categories

Our 43 samples are characterized by wide ranges of alteration extent and alteration type. We assign samples to the following rock types based on macroscopic visual appearance; brown halos, dark gray halos, carbonate veins, iron oxyhydroxide (Fe) stained, breccia, mixed alteration, and minimally altered. The term brown halo refers to all halos that vary from red to brown in color. Similarly, dark gray halo refers to halos ranging from very dark gray to dark green in color. Fe staining includes samples with iron oxyhydroxide staining of secondary minerals or filling of veins and vesicles. Iron oxyhydroxides leave behind a bright red-orange color. We define samples as mixed-alteration when they are visibly altered but without a dominant single alteration type, such as samples with veins and vesicles filled with carbonate, clays and iron oxyhydroxides and have secondary mineral emplacement. Finally, we classify all aphyric samples without visibly altered regions as minimally altered.

Where possible, we analyzed samples of different visibly altered regions within the same basaltic rock to compare the radioactive element concentrations of the different alteration zones. For example, we took two samples from a piece of light gray basalt with a brown halo. For all analyses of alteration halos and carbonate veins, we separated each alteration type from the rest of the rock. Other alteration categories included a mixture of altered rock and background basalt, as they could not be fully separated. We analyzed the 43 samples for their U, Th, and K concentrations.

### U, Th, K Measurements

We used well-established protocols to measure concentrations of U, Th, and K in the 43 samples by inductively coupled plasma-emission spectrometry (ICP-ES) and inductively coupled plasma-mass spectrometry (ICP-MS; Dunlea et al., 2015b). We analyzed these samples at Boston University with a VG PlasmaQuad Excell ICP-MS for U and Th concentrations, and a Jobin-Yvon (JY) Ultima-C ICP-ES for K concentrations. Based on replicate analysis, U and Th precision was 2 and 1%, respectively, of their measured values. The K measurements were within 1% of the measured value. To assess analytical accuracy, we analyzed BHVO-2 Standard Reference Material independently from our calibrations. The measured values agree with the reported accepted values within the analytical precision (Jochum et al., 2005). We list all U, Th, and K data in **Supplementary Table S1**.

### Radiolytic H_2_ Production Model

Our radiolysis model calculates production rates in water near a radionuclide-containing solid (Dzaugis et al., 2015). Previous models, such as those used in sediment (Blair et al., 2007), are not applicable to basalt, because studies of basalt cannot assume homogenous porosity or grain/crystal size smaller than the stopping distance of the radiation. Dzaugis et al. (2015) gives a detailed description of the model that we apply to our samples. In this section, we describe the inputs and assumptions that we used to calculate radiolytic H_2_ production rates in fractures.

There are four dominant parent radionuclides in basalt: ^238^U, ^235^U, ^232^Th, and ^40^K. When these nuclides decay, α, β, and/or γ radiation is emitted. Each type of radiation has different properties that affect radiolytic yield. For example, α particles travel short distances (10s of μm) but have high initial energies and produce the most H_2_ molecules per unit of energy absorbed. Our calculations assume that the entire decay series of ^238^U, ^235^U, and ^232^Th are in secular equilibrium. This assumption is valid for basalt older then 377,000 years (five half-lives of ^230^Th; LaTourrette et al., 1993). However, younger basalt is characterized by isotopic disequilibrium within the ^238^U-decay series, because fractionation during partial melting leads to excess ^230^Th relative to ^238^U (LaTourrette et al., 1993). Due to this ^230^Th enrichment, we slightly underestimate radiolytic H_2_ production rates when we assume secular equilibrium for young basalt.

The distance that α or β radiation travels before losing all of its kinetic energy is called its stopping distance (Spinks and Woods, 1990). Gamma radiation loses energy exponentially with distance and therefore is not assigned a specific stopping distance. Instead, we use the maximum distance traveled by γ-rays to be 10 half-distances, at which point less then 0.1% of their initial energy remains. Stopping distance and γ travel distance depend on the matrix; for example, a 5 MeV α particle will travel about 20 μm in basalt but 40 μm in water. If radiation is emitted from a radionuclide farther from the water interface than the stopping distance, it does not contribute to water radiolysis. Once radiation reaches the basalt-water interface, it continuously ionizes water along its path until it reaches its stopping distance (Le Caër, 2011) or re-enters basalt from the water. Our model incorporates radioactivity, decay energy of each radionuclide, and how each type of radiation attenuates energy along its path. It sums all of the radiation that is absorbed in a fracture. Consequently, the inputs to our model are U, Th, and K concentration, initial energy for all radiation emitted from the isotopes, energy-range relationships for α, β, and γ radiation in basalt and water, H_2_ yield per unit energy for each type of radiation, and distance the radiation travels through water. For the calculations in this paper, we assume there is one centimeter of basalt on either side of the fracture, unless noted otherwise.

We used the data in **Supplementary Table S1** to calculate the radioactivity of our SPG basalt samples for ^238^U, ^235^U, ^232^Th, and ^40^K. We used published radionuclide data (Gale et al., 2013) to calculate the radioactivity of EPR basalt at its time of formation. We used the program RadDecay (Hacker, 1997) to find the initial energy for all radiation emitted from radionuclides in basalt. We calculated stopping distances of α and β particles and half-distances of γ-rays for basalt and water using the energy-range data from the ASTAR, ESTAR (Berger et al., 2005), and X-ray Attenuation (Hubbell and Seltzer, 2004) programs in the NIST database. To determine stopping distances in basalt, we used energy-range data for borosilicate glass (in the NIST database, borosilicate glass is the material with electron density most similar to oceanic basalt). Using these relationships, we developed equations to calculate travel distance given an initial energy. **Supplementary Table S2** summarizes the equations we use in this study.

Using radioactivity, energy and range data, and how many H_2_ molecules are produced per 100 eV (*G*-values), we calculated volume-normalized H_2_ production rates for basalt fractures of widths between 1 um and 1 m. These rates assume (i) basalt of the same composition on both sides of a fracture and (ii) homogeneous distribution of the radionuclides throughout the basalt.

## Results

### South Pacific H_2_ Production Rates

**FIGURE 2:**
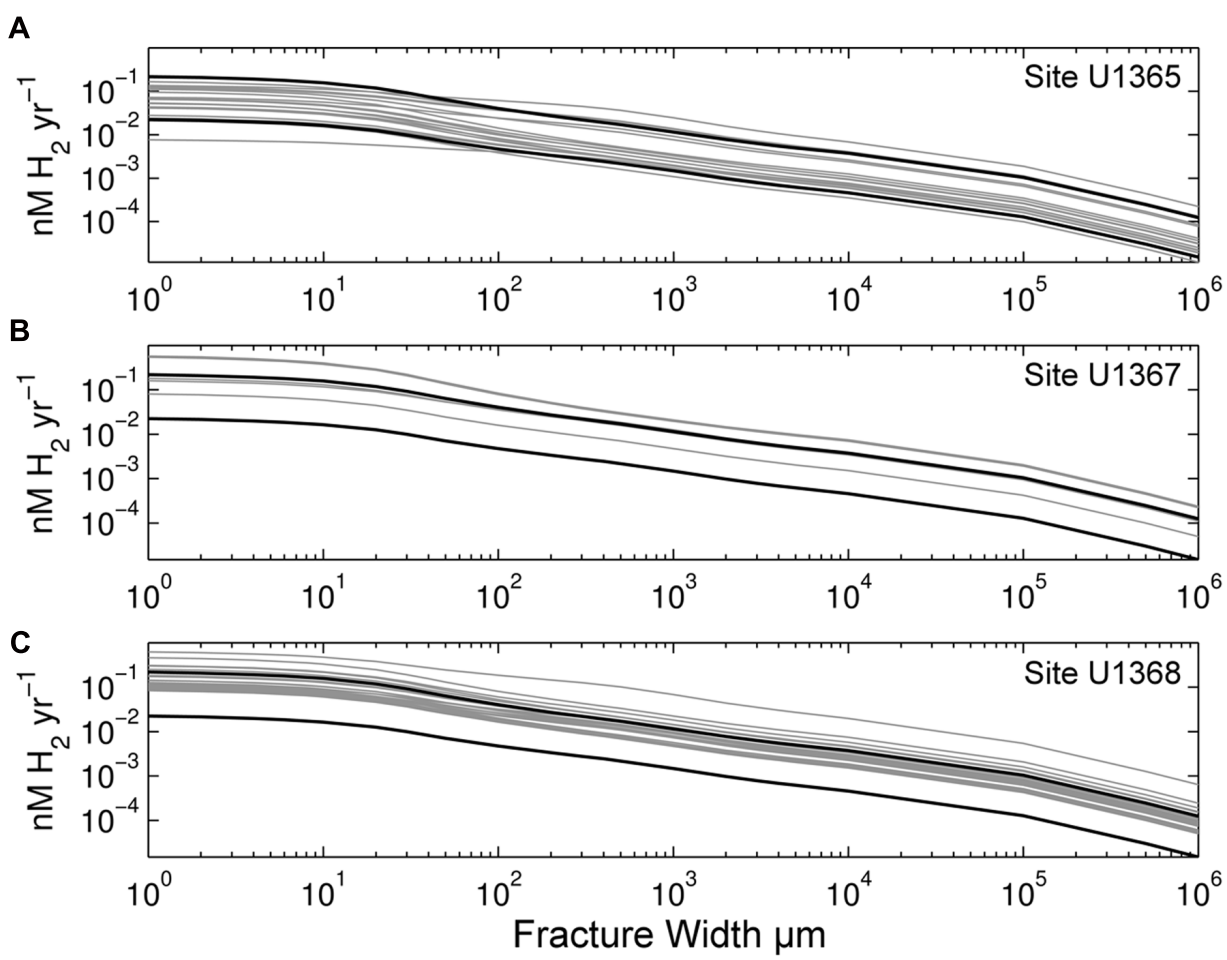
**Radiolytic H_2_ production rates calculated from radionuclide concentrations of our SPG samples.** Volume-normalized rates are shown as a function of fracture width for **(A)** Site U1365, **(B)** Site U1367, and **(C)** Site U1368. Black lines mark maximum and minimum rates calculated from EPR glass samples (Gale et al., 2013). Note that both axes are log scale.

We calculated radiolytic H_2_ production rates as a function of fracture width for all 43 samples. We show the results of these calculations separately for each site in **Figure 2.** Volume-normalized H_2_ production decreases greatly as fracture width increases (from 1μm to 1 m in width; **Figure 2**). For each width, the differences in H_2_ yields between samples are due to variations in radioactive element concentrations. Site U1365 contains the oldest and generally most altered basalt of the three sites (Expedition 329 Scientists, 2011a). It exhibits the lowest H_2_ production rates (**Figure 2A**), while Site U1368, with the youngest basalt age, generally has the highest rates (**Figure 2C**). The SPG sample with the lowest H_2_ production rate (approximately 3X lower then other samples) is from Site U1365 with a Th concentration below the detection limit (0.01 ppm). We calculated the radiolytic H_2_ production rate for this sample using the below detection limit value.

### Radionuclide Compositional Variance

U and K variance in basalt is due to geochemical composition at the time of basalt formation (initial composition) and post-emplacement alteration. In contrast, Th is not significantly modified during post-emplacement alteration (Kelley et al., 2005). We use Th to constrain abundance variations due to initial composition and U/Th ratios to constrain the magnitude of U alteration.

In our samples, Th ranges from below the detection limit (0.01 ppm) to 1.17 ppm. This range is comparable to the entire range of values observed in unaltered basalt glass from the EPR (compositional data from Gale et al., 2013). The U/Th ratios of our samples range from 0.32 to 4.1 (excluding samples with Th below detection limit). This is much greater than ratios observed in unaltered EPR basaltic glass (Gale et al., 2013). However, it is similar to the range in altered material from other studies (e.g., Kelley et al., 2003). About 1/3 of our samples have U/Th ratios that fall within two standard deviations of the mean U/Th ratio in unaltered EPR glass samples. Most of the SPG samples that fall into this category are from the site with youngest basement age, U1368 (11 samples). Only one and two samples at Site U1367 and U1365, respectively, have U/Th ratios indicative of unaltered composition.

### H_2_ Yields and Alteration

We separately describe the calculated radiolytic rates of the three SPG sites because they have different geographic origins, different eruptive histories, and different degrees of deviation from the mean U/Th ratio of unaltered EPR glass. Site U1365 exhibits a 21-fold range in rates, whereas Sites U1367 and U1368 show 7- to 8-fold ranges. Overall, Site U1365 has the lowest rates, while Site U1368 has the highest (**Figure 2**).

**FIGURE 3:**
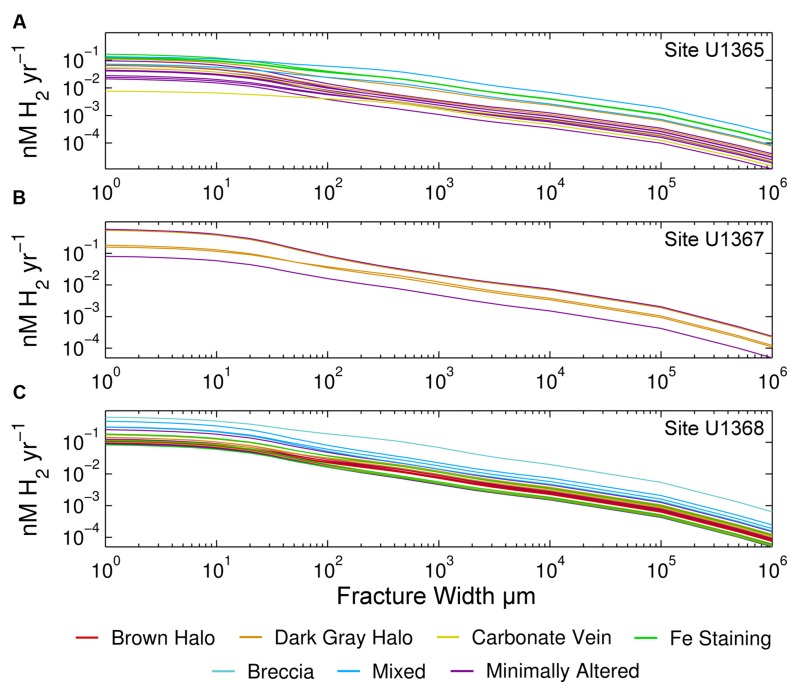
**Relation of rock alteration to radiolytic H_2_ production rates.** Volume-normalized rates are given for Sites **(A)** U1365, **(B)** U1367, **(C)** U1368. Colors show the alteration type to which we assigned each sample. Note that both axes are log scale. As in **Figure 2**, rates are given as a function of fracture width.

Our samples show no clear relationship between alteration type and H_2_ production rates. The H_2_ production rates calculated for all alteration categories, including minimally altered samples, seem to span the entire range; no alteration type exhibits a significantly different range of H_2_ production rates than the others (**Figure 3**). There is some separation of alteration types within the individual sites. At Site U1365, iron stained samples tend to have higher rates, while at Site U1368 the mixed alteration samples and the breccia are highest (**Figure 3**). Two of the three mixed alteration samples at U1368 have U/Th ratios within the unaltered range indicating that the high range may not completely be due to alteration. Many of the H_2_ production rates for the different samples overlap; the rates calculated for each sample at the basalt-water interface are given in **Supplementary Figure S1**.

To further investigate the impact of alteration, we calculate the *U* due to alteration (*U_alt_*) as

$$U_{alt}=U_{meas}-{\left(\frac{U}{Th}\right)}_{unalt}*{Th}_{meas}$$

where the subscript *meas* refers to measured values and ${\left(\frac{U}{Th}\right)}_{unalt}$ is the average ratio in unaltered EPR basalt (0.37 ± 0.08). We then calculate H_2_ yield due to *U_alt_* and determine its fractional contribution to the total yield. There are 27 samples that have excess U that is significantly (more then two standard deviations) different than zero. For these samples, the H_2_ yield based on *U_alt_* ranges from 0 to 4.9 × 10^-1^ ± 1.5 × 10^-2^nM H_2_ yr^-1^, and can contribute up to 85 ± 3% of the radiolytic H_2_ produced (average of 31 ± 7% for all samples with altered U/Th ratios).

### Influence of Fracture Width and Basalt Width on H_2_ Production Rates

While compositional variation leads to a large range of radiolytic H_2_ production rates (almost two orders of magnitude within the SPG samples), fracture width has an even greater effect on volume-normalized H_2_ production (moles per vol. of water per time). The production rate per volume of water decreases as fracture width increases. This decline in volume-normalized rates is most pronounced after α and β particles run out of energy. To illustrate this effect, we calculated volume-normalized production rates for a range of fracture widths that occur in basement basalt (1 μm to 1 m). Volume-normalized H_2_ production rates are highest in microfractures (<10 μm), regardless of radionuclide concentration, and strongly decrease as fracture width increases (**Figure 2**). Production rates in 1 μm wide fractures differ by more than three orders of magnitude from rates in 1 m wide fractures. Volume-normalized H_2_ production rates are highest at the rock–water interface, due to high dose rates. However, if production rate is normalized to the surface area of fractures, it increases with fracture width as more radiation, especially from γ-rays, is absorbed in wider fractures than narrower fractures.

**FIGURE 4:**
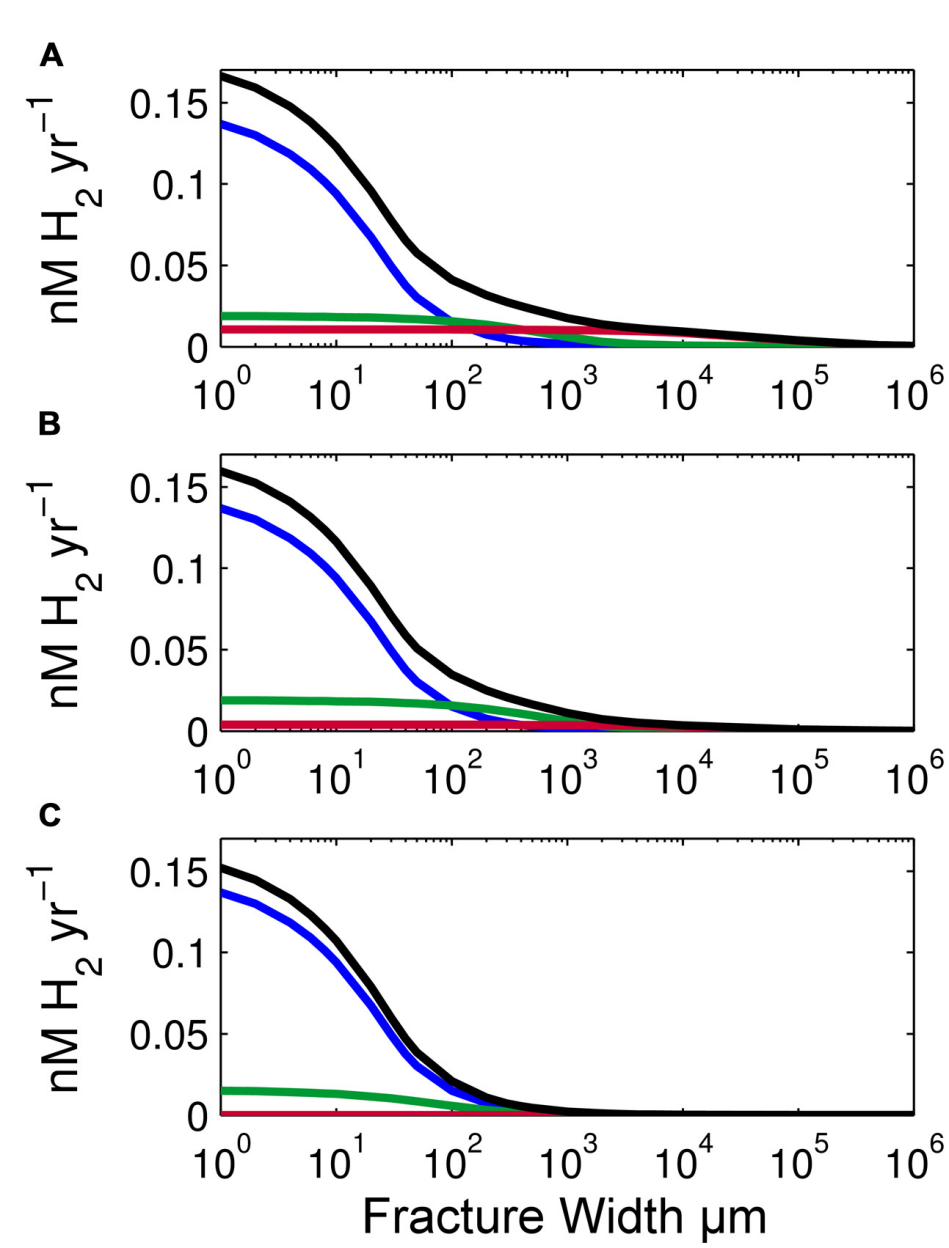
**Effect of basalt thickness on radiolytic H_2_ production rates.** **(A)** Volume-normalized radiolytic H_2_ production for 1-m thick basalt using an average SPG composition of our samples, **(B)** 1-cm thick basalt, and **(C)** 100-μm thick basalt. Total production rates are shown as black lines. Rates due to α radiation are shown in blue, β radiation in green and γ radiation in red. *X*-axis is log scale.

Radiolytic H_2_ production rates also vary with the thickness of basalt that abuts a fracture (**Figure 4**). To illustrate this effect, we calculated production rates based on a single SPG sample and three different thicknesses: 1 m, 1 cm and 100 μm. Thickness affects the amount of radiation emitted to water. One meter of basalt is approximately equivalent to an infinite basalt thickness because less than 0.1% of the radiation travels beyond a meter (10 half-distances of γ-rays); the amount of energy that reaches the water approaches its maximum at about a meter of basalt.

This dependence on basalt thickness is clearly illustrated by comparing H_2_ production by β and γ radiation with 100 μm thick basalt (**Figure 4C**) to H_2_ production by β and γ radiation with 1 cm thick or 1 m thick basalt (**Figures 4A,B**). The decrease in thickness from 1 m to 100 μm of basalt causes a 20% decrease in β-produced H_2_ and a 98.9% decrease in γ-produced H_2_. Because the stopping distance for β and γ radiation is much greater than 100 μm in basalt, the change in thickness greatly impacts the number of β particles and γ-rays emitted from the basalt. Despite these effects of basalt thickness on H_2_ production by β and γ radiation, total radiolytic H_2_ production rate is not greatly affected by basalt thickness because α radiation is responsible for most radiolytic H_2_ production in our examples (**Figure 4**). Total H_2_ production rate is only 4% less with 1 cm of basalt and 10% less with 100 μm than 1 m of basalt. In all three cases, α-dose rate is the same, because α particles penetrate no more than 50 μm of basalt (25 μm on average). Therefore, in all three scenarios, all the α radiation that can reach fracture water does.

## Discussion

In this section, we discuss (i) factors that impact radiolytic H_2_ production rates, (ii) their potential importance to electron donor fluxes in subseafloor basalt, and (iii) the number of cells that might be supported by rates of radiolytic H_2_ production in representative fractures. We first consider the fractured nature of oceanic basement and how both fracture width and basalt thickness affect radiolysis rates. We address how source-melt composition and subsequent low-temperature alteration play a role in determining the distribution of radionuclides in our sample set and rates of radiolytic H_2_ production. We then compare electron donor fluxes from radiolytic H_2_ production and from iron oxidation. Finally, we estimate numbers of cells that might be supported by radiolysis in SPG basaltic fractures, based on comparison to per-cell O_2_ consumption rates in SPG sediment.

### Factors that Influence H_2_ Production

We focused this study on radiolysis in fractures because most of the water in oceanic basement resides in, and travels through, fractures (Fisher, 1998). The three primary categories of porosity in oceanic crust are (i) macroscopic features, such as lithological boundaries (e.g., surfaces of lava flows), voids associated with pillow basalts, and collapse structures; (ii) microcracks which have narrow widths and limited lateral extent, and (iii) vesicles and other primary porosity, which we do not consider since they are typically disconnected and isolated. Of these three categories, the first is most closely related to formation-scale permeability (Fisher, 1998). Fluid flow in the igneous basement is mostly through interconnected fractures in the oceanic basement (Wang, 2004), e.g., through fractures between pillow basalts and along the margins of lava flows. This relationship between fluid flow and interconnected fractures is apparent in the distribution of alteration halos, which are mainly associated with veins in the basalt, indicating that chemical transport is very limited in the low porosity, impermeable basalt matrix (Bach and Edwards, 2003).

Our focus on quantifying radiolytic rates in fractures differs from previous studies of radiolysis in continental crust (e.g., Lin et al., 2005a), which assume porosity to be homogenously distributed throughout the rock. Such an assumption is very problematic for estimating radiolytic H_2_ production in hard-rock aquifers, where most porosity is in heterogeneously distributed fractures and much of the radiation emitted from radionuclides in the rock will never reach water. As we discussed in Section “Results,” properties that most affect H_2_ production rates in hard-rock aquifers include fracture width, rock thickness and rock composition. Of these properties, fracture width and rock composition are the most important.

Fracture width greatly influences radiolytic H_2_ production rates (**Figures 2–4**) because volume-normalized H_2_ production rapidly decreases with distance from the rock-water interface. This decrease in H_2_ production is due to the limited ranges of α and β radiation and increased volume of water. Of the three types of radiation, α radiation has the highest initial energy and highest *G*-value at 1.2 molecules H_2_ per 100 eV (Pastina and LaVerne, 2001). However, it also has the shortest range. Consequently, H_2_ production by α radiation extends only several tens of microns into fracture water. Beta and gamma radiation have lower *G*-values (0.6 and 0.45 molecules H_2_ 100 eV^-1^, respectively) than α particles (Kohan et al., 2013; Mustaree et al., 2014), but β particles can travel 1000s of μm in water and γ-rays travel for tens of cm. Consequently, α radiation dominates total radiolytic H_2_ production near the rock-water interface, while γ-produced H_2_ is highest in fractures greater than 1 cm (**Figure 4**). Due to the very high rate of H_2_ production from α radiation, volume-normalized H_2_ production rates in 1 μm fractures are on average 190 times higher than fractures 10 cm in width and 1.6 × 10^3^ times higher than in 1 m fractures. In short, microfractures are hotspots of radiolytic H_2_ production.

To a much lesser extent, total radiolytic H_2_ production varies with the thickness of the abutting rock (**Figure 4**). Over a four-order magnitude of change in basalt thickness (from 100 μm to 1 m of basalt), H_2_ production rates change by only 10% at the rock–water interface. This change is due to differences in the absorbed doses of β and γ radiation. This change is small because α radiation from deeper in the rock does not penetrate to the surface. If the thickness of basalt facing a fracture is less than 50 μm, the rates will drop more dramatically due to a lower absorbed α-dose rate.

Along with microfractures, high concentrations of U, Th, and K create hotspots of radiolytic H_2_ production. Uranium, Th, and K concentrations differ widely from sample to sample within and between the three sites. To illustrate the range of H_2_ yields due to initial composition variance, we show a H_2_ production range for unaltered basaltic EPR glass samples (Gale et al., 2013). The range of calculated H_2_ production at the basalt-water interface is approximately 10-fold for the group of EPR samples with Th composition within two standard deviations of the mean (∼90% of the samples) (black lines, **Figure 2**). The range is much greater (93-fold) if we consider all EPR glass measurements reported by Gale et al. (2013), which include measurements of enriched mid-ocean ridge basalt (E-MORB) that have much higher U and Th concentrations. Basement comprised of E-MORB will have higher radiolytic yields than normal ocean basalt.

The 10-fold difference in H_2_ yields from EPR data suggests that basaltic source-melt composition has a significant effect on radiolytic rates. Given the wide range of compositions exhibited by the EPR samples, we expect variation in radiolytic H_2_ production rates from one SPG site to another, as they all have different geographic locations and ages of origin (**Figure 1**). In addition, the basalt at Site U1365 has a very different eruption history than the basalt at Sites U1367 and U1368. The basalt drilled at Site U1365 was accreted during medium to fast spreading and is comprised mostly of large sheet flows. In contrast, the basalts drilled at Sites U1367 and U1368 are predominantly pillow basalt, and were likely produced during slower spreading than at Site U1365 (Zhang et al., 2013; Zhang and Smith-Duque, 2014).

Site U1365 has lower radiolytic H_2_ production rates than Sites U1367 and U1368, despite having the oldest and most altered basalt (**Figure 2**). This can be attributed to low initial concentrations, as indicated by Th values. Thorium concentrations of U1365 samples are all below 0.2 ppm. These concentrations are lower than those in the samples from the other localities and fall into the bottom half of EPR Th data from Gale et al. (2013). Even with enrichment of U during alteration (indicated by high U/Th ratios) at Site U1365, the radiolytic rates are low compared to the other localities indicating that they are greatly affected by initial radioisotope concentrations. This variation in source-melt composition is consistent with the results of Zhang and Smith-Duque (2014), who used differences in initial composition to explain geochemical variance between Sites U1365 and U1368. In addition, there is also likely vertical variation at each site due to changes in source composition between eruptive events (e.g., Bergmanis et al., 2007). Zhang et al. (2013) reported some vertical variation at Site U1368. Using U, Th and K from Zhang et al. (2013) results in a 6X difference in radiolytic rates between their lowest normal-MORB sample and the sample they suggest was influenced by an enriched-MORB source. This difference is similar to the sevenfold range in rates calculated from our radioisotope concentration data for this site.

Concentrations of U and K often increase with alteration (e.g., Staudigel et al., 1996; Teagle et al., 1996; Kelley et al., 2003). We use U/Th ratios to constrain how much excess U a sample contains. We don’t calculate excess values for K, because K/Th ratios in fresh basalt are not consistent enough to calculate excess K in this manner. Potassium is typically concentrated in such alteration minerals as smectite and zeolite, as well as K-feldspar (Alt and Teagle, 2003; Bartetzko, 2005). Shipboard logs of natural gamma radiation (NGR) show clear evidence of such K concentration at alteration fronts in Site U1365 basalt (D’Hondt et al., 2011, 2013). However of the three radionuclides, K contributes the least to total H_2_ production because it only produces β and γ radiation when it decays. Using an average concentration for the SPG samples, K contributes 13% to total production at the basalt–water interface. Uranium and Th decay series account for 65 and 22% of the H_2_ produced, respectively. Of our SPG basalt samples, K decay dominates H_2_ production in only one sample (U1365E-3R-4W 25/30), where it accounts for 48% of the total H_2_ yield.

The U decay series is overwhelmingly the largest contributor to H_2_ production. Uranium enrichment often occurs in carbonate veins and at oxidation/reduction fronts during basalt alteration (Alt et al., 1992; Farr et al., 2001; Kelley et al., 2003). Twenty-seven of our SPG samples exhibit U/Th ratios that indicate U enrichment, excluding the sample with Th below the detection limit. In these twenty-seven samples, U enrichment ranges up to a 10X increase, with an average U/Th ratio indicating a 2X increase in U. Our samples bracket the typical enrichment value of 5X for altered basalt when compared to unaltered glass (Kelley et al., 2003). The samples with excess U increase H_2_ yields on average by 29 ± 6%, 60 ± 3%, and 23 ± 10% at Sites U1365, U1367, and U1368, respectively. Therefore, the variation in radiolytic H_2_ production rates that we calculate for bulk basalt likely accounts for much of the variation that would be seen in the oceanic crust.

As shown by the variety of visual alteration categories and the wide range of U/Th ratios exhibited by our SPG samples, the kind, and extent of alteration vary greatly on a variety of spatial scales. The most intensely altered samples are likely those with most U enrichment; however that does not mean that they have the highest radionuclide concentrations. This can make links between alteration extent and radiolytic yields difficult to clearly identify. Separation of samples by alteration category or by U/Th ratios does not result in obvious patterns, suggesting that there is no simple relationship between radiolytic rates and alteration. Visual inspection of the samples is not enough to fully assess the impact of alteration on radiolytic rates. For example, some of the SPG samples with brown and dark gray alteration halos have U/Th ratios that indicate no U enrichment. Other studies, such as the SPG study of Zhang and Smith-Duque (2014) and the western Pacific study of Kelley et al. (2003) have shown U enrichment especially within these zones. Much closer analysis of mineral and chemical composition would be needed to fully assess effects of alteration on radionuclide concentrations and radiolytic H_2_ production rates of SPG basalt.

The rates we calculate are based on bulk-rock analyses and may underestimate radiolytic H_*2*_ production associated with specific alteration phases. For localized mineral phases with high U, Th, or K concentrations rates will be much higher. Staudigel et al. (2008) suggest that weathering of basaltic glass is the dominant process of chemical exchange between basalt and seawater. Alteration of basaltic glass produces palagonite (Staudigel et al., 2008; Türke et al., 2015), a very porous mineral (∼14–38 wt% H_*2*_O; Pauly et al., 2011) with high concentrations of radioactive elements (Türke et al., 2015).

Palagonite is of special interest for studies of radiolytic H_2_ production in subseafloor basalt because its porous nature and its enriched radionuclide concentrations must result in high radiolytic rates (Türke et al., 2015). Türke et al. (2015) calculated H_2_ production rates within palagonite rims and show that H_2_ can accumulate to minimum concentrations needed for hydrogentrophy at North Pond (on the western flank of the Mid-Atlantic Ridge). For their calculations, Türke et al. (2015) used a porosity model based on protocols from Lin et al. (2005a) and Blair et al. (2007). Because palagonite is so porous, these models’ assumptions of fine grain size and homogeneously distributed porosity are broadly relevant for palagonite calculations. However, these models’ assumption that all of the radiation interacts with water is not appropriate for relatively thin palagonite occurrences. Türke et al. (2015) note this issue and calculate yields assuming 1% efficiency. However, there is no explicit way to calculate the actual efficiency using their model. The average H_2_ production rates calculated by Türke et al. (2015) are about four times higher than average SPG rates in microfractures. Consequently, palagonite and other similarly porous alteration minerals may significantly increase radiolytic H_2_ production relative to neighboring basalt.

Calculating the global rate of radiolytic H_2_ production in oceanic basement requires firm constraints on the distribution of fracture widths in the basement, mean concentrations of U, Th and K in the basalt, and porosity and abundance of alteration minerals in the basement. Mapping the horizontal and vertical distribution of radiolytic H_2_ production rates in subseafloor basalt requires information beyond the mean, including knowledge of horizontal and vertical variation in fracture width and number, radionuclide (U, Th, and K) concentrations, and porosity of alteration phases. Spatial variation in radionuclide concentrations is especially difficult to constrain, because it in turn depends on both (i) initial composition (which can vary from region to region, and even between successive eruptive events at the same location; Bergmanis et al., 2007) and (ii) alteration history (including the extent and kinds of secondary mineralization; Alt, 2004).

### Comparison of Electron Donor Fluxes from Radiolytic H_2_ Production and Iron Oxidation

**FIGURE 5:**
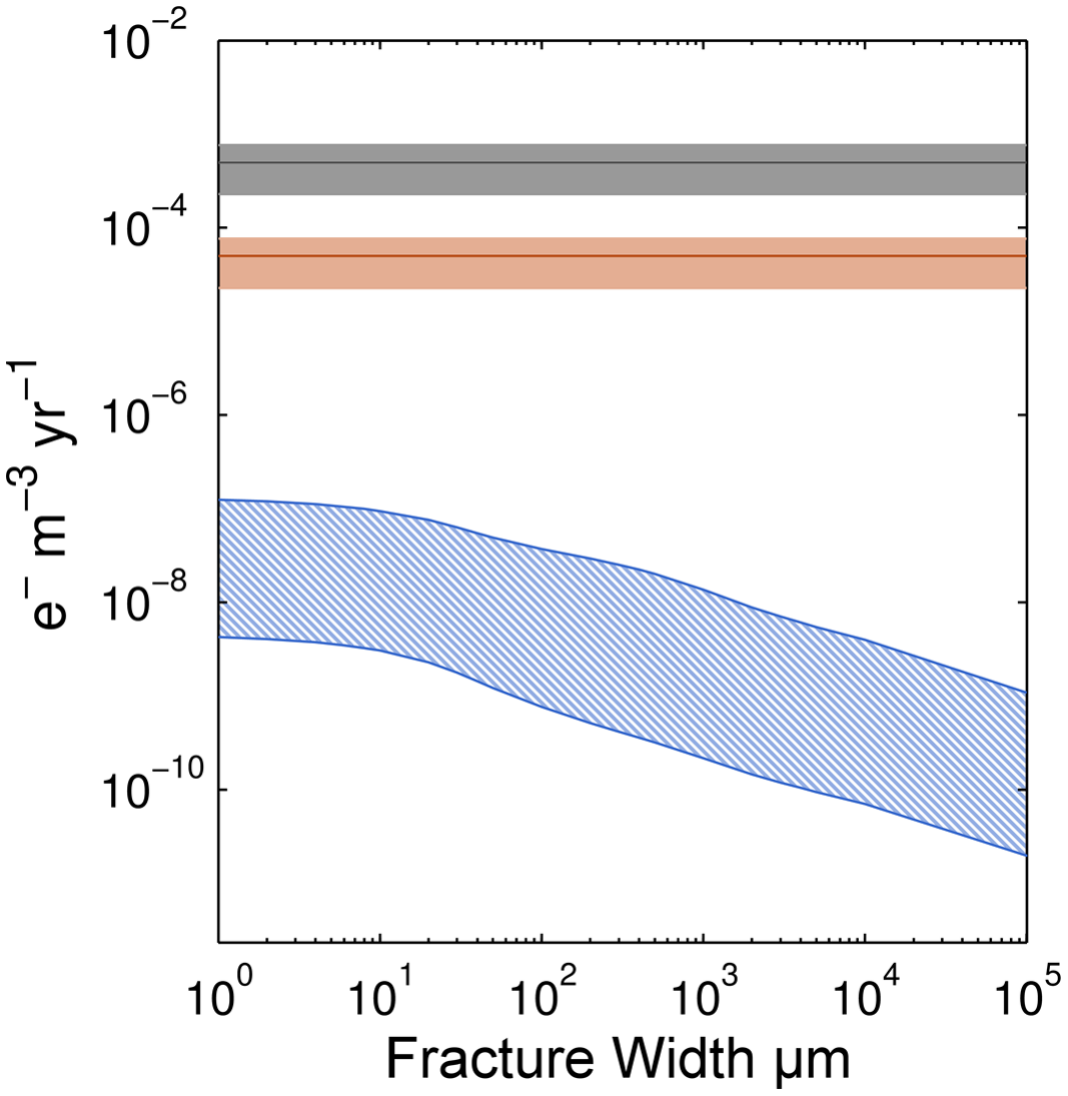
**Comparison of electron transfer rates supportable by Fe oxidation and radiolytic H_2_ production.** Ranges of electron (e^-^) transfer rates due to Fe oxidation are shown for basalt of two different ages, 1 and 10 Ma shown by the gray and orange shading, respectively. The blue-hatched region indicates the range of electron transfer supportable by radiolytic H_2_ production.

To assess the potential importance of radiolytic H_2_ production for microbial communities in subseafloor basalt, we compare our calculated radiolytic H_2_ production rates to iron oxidation rates calculated from Fe(III)/ΣFe ratios compiled by Bach and Edwards (2003; **Figure 5**).

To compare these rates, we convert them to electron transfer rates per unit volume. For Fe oxidation, one electron is transferred during oxidation of Fe(II) to Fe(III); for H_2_, two electrons are transferred for each H_2_ molecule produced. For direct comparison, we assume a cubic meter of basalt with 10% porosity, density of 2950 kg/m^3^ and 8 wt.% Fe (Bach and Edwards, 2003). We use an initial Fe(III)/ΣFe ratio of 0.15 ± 0.05 for fresh basalt and a final ratio of 0.45 ± 0.15 for basalt greater than 10 Ma (Bach and Edwards, 2003). These values include the variation associated with the average Fe(III)/ΣFe ratios at their respective basalt ages. Given these ratios, we calculate a mean oxidation rate of 0.19 ± 0.1 mol Fe oxidized kg basalt^-1^ Myr^-1^ for the first 10 Myr of the basalt’s existence. Considerable variability in the Fe(III)/ΣFe ratios given by Bach and Edwards (2003) indicates that different regions within the basement have higher, or lower, Fe oxidation rates. For H_2_ calculations, we assume that each unique fracture width makes up the 10% porosity in the basalt. For example, there are one thousand 100 μm fractures or one 10 cm fracture in our calculations to account for the correct water-rock ratio.

Given these assumptions, for oceanic crust younger than 10 Ma, calculated electron transfer rates (mol e^-^ per m^3^ rock per year) due to Fe oxidation are about three orders of magnitude higher than electron transfer rates associated with radiolytic H_2_ production (**Figure 5**). However, after circa 10 Ma, mean Fe(III)/ΣFe ratios appear to plateau, suggesting that all available Fe has been oxidized (Bach and Edwards, 2003). This means that Fe oxidation rates in basalt older than circa 10 Ma are near zero. In this older basalt, radiolytic H_2_ may be more important than Fe(II) as an electron donor for microbial communities.

This change from Fe(II) to radiolytic H_2_ as the predominant electron donor is consistent with the palagonite-based conclusion of Türke et al. (2015). In young ridge flanks, Fe oxidation is a main source of energy for microorganisms due to interaction of seawater with fresh basalt in fractures. However, in older crust, after permeability decreases and access to fresh basalt is prevented by alteration phases, the availability of Fe decreases and the dominant electron donor may be radiolytic H_2_ (Türke et al., 2015).

### Numbers of Cells that Might be Supported by Radiolytic H_2_ Production in Fractures

To estimate how many cells might be supported by oxidation of radiolytic H_2_, we divide the radiolytic H_2_ flux for different fracture widths by the net per-cell oxygen reduction rates in SPG sediment (**Table 1**). As with our comparison to Fe(II) oxidation, we convert both radiolytic H_2_ production rates and oxygen reduction rates to electron transfer rates. Net per-cell oxygen reduction rates in subseafloor SPG sediment range between 4.2 × 10^-17^ ± 2.6 × 10^-17^ mol e^-^ cell^-1^ yr^-1^ and 6.8 × 10^-15^ ± 1.3 × 10^-15^ mol e^-^ cell^-1^ yr^-1^ (D’Hondt et al., 2015). Using these maximum and minimum oxygen reduction rates and the radiolytic electron fluxes (mol e^-^ cm^-2^ yr^-1^) for discrete fracture widths in our least and most radioactive basalt samples, we can estimate the number of cells that might be supported by H_2_ production. If the cells respire at the same rate as the aerobic communities in subseafloor SPG sediment, radiolytic H_2_ production in the volume of water adjacent to a square cm of the least radioactive SPG basalt might support up to 30 cells. H_2_ production in the same volume of water adjacent to a square cm of the most radioactive SPG basalt might support up to 1500 cells.

**Table 1:** Numbers of cells that might be supported by radiolytic H_2_ production in SPG basalt.

	Least radioactive SPG basalt sample		Most radioactive SPG basalt sample	
		
Fracture Width (μm)	e^-^ flux per area of fracture surface (mol e^-^/cm^2^/yr)	Number of cells that might be supported by this flux	e^-^ flux per area of fracture surface (mol e^-^/cm^2^/yr)	Number of cells that might be supported by this flux
10^0^	7.65E-19	1.1E-04 ± 2E-05 to1.8E-02 ± 1E-02	6.25E-17	9.2E-03 ± 2E-03 to1.5E+00 ± 9E-01
10^1^	6.50E-18	9.6E-04 ± 2E-04 to1.6E-01 ± 1E-01	4.72E-16	7.0E-02 ± 1E-02 to1.1E+01 ± 7E+00
10^2^	3.82E-17	5.6E-03 ± 1E-03 to9.2E-01 ± 6E-01	1.86E-15	2.8E-01 ± 5E-02 to4.5E+01 ± 3E+01
10^3^	1.08E-16	1.6E-02 ± 3E-03 to2.6E+00 ± 2E+00	6.80E-15	1.0E+00 ± 2E-01 to1.6E+02 ± 1E+02
10^4^	3.53E-16	5.2E-02 ± 1E-02 to8.5E+00 ± 5E+00	1.99E-14	2.9E+00 ± 5E-01 to4.8E+02 ± 3E+02
10^5^	9.90E-16	1.5E-01 ± 3E-02 to2.4E+01 ± 1E+01	5.45E-14	8.1E+00 ± 1E+00 to1.3E+03 ± 8E+02
10^6^	1.16E-15	1.7E-01 ± 3E-02 to2.8E+01 ± 2E+01	6.35E-14	9.4E+00 ± 2E+00 to1.5E+03 ± 1E+03


*Electron fluxes are calculated from radiolytic H_2_ production rates for 1 sq. cm of basalt for the least radioactive and most radioactive SPG basalt samples. The ranges of cell numbers that might be supported are calculated from minimum and maximum O_2_ consumption rates that are distinguishable from zero within subseafloor SPG sediment (D’Hondt et al., 2015).*

## Conclusion

The extent of life in oceanic crust must depend in large part on the availability of electron donors and acceptors. Water radiolysis produces H_2_, which can be metabolized by microorganisms. Microfractures and local maxima in radionuclide concentrations serve as hotspots for radiolytic H_2_ production and may also act as hotspots for microbial life. Differences in initial melt composition and low-temperature alteration by seawater affect concentrations and distributions of U, Th, and K within the basement, and consequently also change radiolytic H_2_ production rates. Our calculations suggest that in young (less than 10 Ma) basalt, oxidizable Fe(II) is a far more abundant electron donor than radiolytic H_2_. However, in older seafloor, where little Fe is accessible to oxidants in the formation water, radiolytic H_2_ may be the dominant electron donor. Radiolytic H_2_ in the water adjacent to a square cm of SPG basaltic fracture may support up to 10^3^ cells if the cells respire at the same rate as net per-cell oxygen reduction in subseafloor SPG sediment.

## Author Contributions

All authors listed, have made substantial, direct, and intellectual contribution to the work, and approved it for publication.

## Conflict of Interest Statement

The authors declare that the research was conducted in the absence of any commercial or financial relationships that could be construed as a potential conflict of interest.
