# Supplementary material for: Radiolytic Hydrogen Production in the Subseafloor Basaltic Aquifer
Source: Front Microbiol. 2016 Feb 4;7:76. doi: 10.3389/fmicb.2016.00076 (PMC4740390; doi:10.3389/fmicb.2016.00076)
Supplement: Supplementary file 1 [file Table_1.PDF]

Supplementary Material

Radiolytic hydrogen production in the subseafloor basaltic aquifer

Mary E. Dzaugis*, Arthur J. Spivack, Ann G. Dunlea, Richard W. Murray and Steven D'Hondt

* Corresponding Author: mdzaugis@my.uri.edu

Table S1. Radionuclide concentration of SPG basalt from IODP Expedition 329. This list includes K, Th and U concentrations for the 43 samples as well as their designated alteration type.

Site	Hole	Core	Sect.	Interval Top	Interval Bot	Description	$K_2 0$ (wt.%)	Th (ppm)	U (ppm)
U1365	E	3R	1-W	51	54	Minimally Altered	0.079	0.103	0.052
U1365	E	3R	3-W	40	44	Carbonate Vein	0.110	0.006*	0.005
U1365	E	3R	3-W	40	44	Minimally Altered	0.080	0.069	0.030
U1365	E	3R	4-W	25	30	Mixed	1.550	0.191	0.098
U1365	E	5R	1-W	50	54	Minimally Altered	0.099	0.100	0.067
U1365	E	5R	3-W	13	17	Mixed	0.526	0.106	0.073
U1365	E	6R	1-W	14	16	Minimally Altered	0.044	0.143	0.142
U1365	E	6R	2-W	40	45	Minimally Altered	0.055	0.094	0.051
U1365	E	6R	2-W	108	111	Minimally Altered	0.033	0.057	0.024
U1365	E	8R	1-W	58	63	Carbonate Vein	0.024	0.001*	0.096
U1365	E	8R	1-W	58	63	Minimally Altered	0.089	0.041	0.029
U1365	E	9R	3-W	130	133	Minimally Altered	0.061	0.128	0.088
U1365	E	11R	1-W	80	86	Dark Gray Halo	0.321	0.178	0.143
U1365	E	12R	2-W	39	45	Fe Staining	0.738	0.170	0.131
U1365	E	12R	2-W	39	45	Fe Staining	0.753	0.163	0.145
U1365	E	12R	2-W	117	122	Fe Staining	0.639	0.163	0.223
U1365	E	12R	4-W	31	34	Dark Gray Halo	0.093	0.174	0.080
U1367	F	2R	3-W	35	39	Dark Gray Halo	0.224	0.220	0.092
U1367	F	2R	3-W	69	74	Dark Gray Halo	0.582	0.203	0.204
U1367	F	2R	3-W	69	74	Minimally Altered	0.241	0.238	0.982
U1367	F	5R	1-W	40	43	Dark Gray Halo	0.392	0.231	0.250
U1367	F	6R	1-W	80	85	Minimally Altered	0.175	0.209	0.089
U1368	F	2R	1-W	33	36	Mixed	0.397	0.800	0.347
U1368	F	2R	1-W	72	76	Fe Staining	0.434	0.557	0.182
U1368	F	2R	2-W	88	92	Mixed	0.644	1.173	0.541
U1368	F	2R	3-W	57	62	Mixed	0.665	0.857	0.327

U1368	F	2R	4-W	102	107	Minimally Altered	0.496	0.769	0.254
U1368	F	4R	1-W	71	75	Dark Gray Halo	0.531	0.292	0.216
U1368	F	5R	2-W	43	48	Minimally Altered	0.116	0.293	0.140
U1368	F	7R	2-W	24	28	Minimally Altered	0.361	0.304	0.168
U1368	F	7R	2-W	24	28	Dark Gray Halo	0.415	0.285	0.166
U1368	F	9R	1-W	42	47	Fe Staining	0.183	0.242	0.086
U1368	F	10R	2-W	117	120	Minimally Altered	0.317	0.301	0.141
U1368	F	10R	2-W	117	120	Dark Gray Halo	0.589	0.264	0.113
U1368	F	12R	2-W	87	91	Dark Gray Halo	0.157	0.270	0.116
U1368	F	12R	4-W	21	24	Fe Staining	0.161	0.260	0.142
U1368	F	13R	2-W	0	5	Minimally Altered	0.136	0.252	0.109
U1368	F	13R	2-W	0	5	Brown Halo	0.317	0.240	0.111
U1368	F	13R	2-W	31	34	Minimally Altered	0.151	0.241	0.101
U1368	F	13R	2-W	31	34	Brown Halo	0.355	0.245	0.106
U1368	F	13R	2-W	57	59	Minimally Altered	0.146	0.242	0.101
U1368	F	13R	2-W	57	59	Brown Halo	0.424	0.230	0.115
U1368	F	14R	1-W	111	114	Breccia	3.720	0.243	0.822

*Sample measurement below detection limit of 0.01 ppm
