# Supplementary material for: Radiolytic Hydrogen Production in the Subseafloor Basaltic Aquifer
Source: Front Microbiol. 2016 Feb 4;7:76. doi: 10.3389/fmicb.2016.00076 (PMC4740390; doi:10.3389/fmicb.2016.00076)
Supplement: Supplementary file 2 [file Table_2.PDF]

frontiers

## *Supplementary Material*

# Radiolytic hydrogen production in the subseafloor basaltic aquifer

**Mary E. Dzaugis*, Arthur J. Spivack, Ann G. Dunlea, Richard W. Murray and Steven D'Hondt**

* Corresponding Author: mdzaugis@my.uri.edu

**Table S2. Energy-Range equations and b-values.** We calculate the travel distance (µm) in water and borosilicate glass ('Pyrex') for α and β particles using the formula $\text{Distance} = h * E^b$ for the energy ranges indicated. b-values are also used in the radiolysis model to account for attenuation of α and β particles while traveling (see Dzaugis *et al.*, 2015). To calculate the attenuation coefficients for γ-rays at any initial energy, we fit a polynomial to the attenuation coefficient (µ)-energy (E) data from the NIST database. In the equation below, y is log(µ) and x is log(E).

| Radiation | Material | Energy Range (MeV) | h | b value |
|---|---|---|---|---|
| Alpha | 'Pyrex' | 3.00 – 9.00 | 2.2 | 1.5 |
| | Water | 2.00 – 9.00 | 3.6 | 1.5 |
| | | < 2.00 | 5.5 | 0.81 |
| Beta | 'Pyrex' | 1.00E-02 – 4.50E-01 | 3.4E03 | 1.7 |
| | | 4.50E-01 – 4.50E00 | 2.2E03 | 1.07 |
| | Water | 1.00E-02 – 4.50E-01 | 6.7E03 | 1.7 |
| | | 4.50E-01 – 4.50E00 | 4.2E03 | 1.2 |
| Gamma | 'Pyrex' | Equation: $y = 0.012x^5 + 0.15x^4 - 0.050x^3 - 0.19x^2 - 0.42x - 4.8$ | | |
| | Water | Equation: $y = -0.037x^5 + 0.070x^4 + 0.11x^3 - 0.14x^2 - 0.51x - 5.146$ | | |
