# Supplementary material for: Radiolytic Hydrogen Production in the Subseafloor Basaltic Aquifer
Source: Front Microbiol. 2016 Feb 4;7:76. doi: 10.3389/fmicb.2016.00076 (PMC4740390; doi:10.3389/fmicb.2016.00076)
Supplement: Supplementary file 3 [file Image_1.PDF]

Supplementary Material

Radiolytic hydrogen production in the subseafloor basaltic aquifer

Mary E. Dzaugis*, Arthur J. Spivack, Ann G. Dunlea, Richard W. Murray and Steven D'Hondt

* Corresponding Author: mdzaugis@my.uri.edu

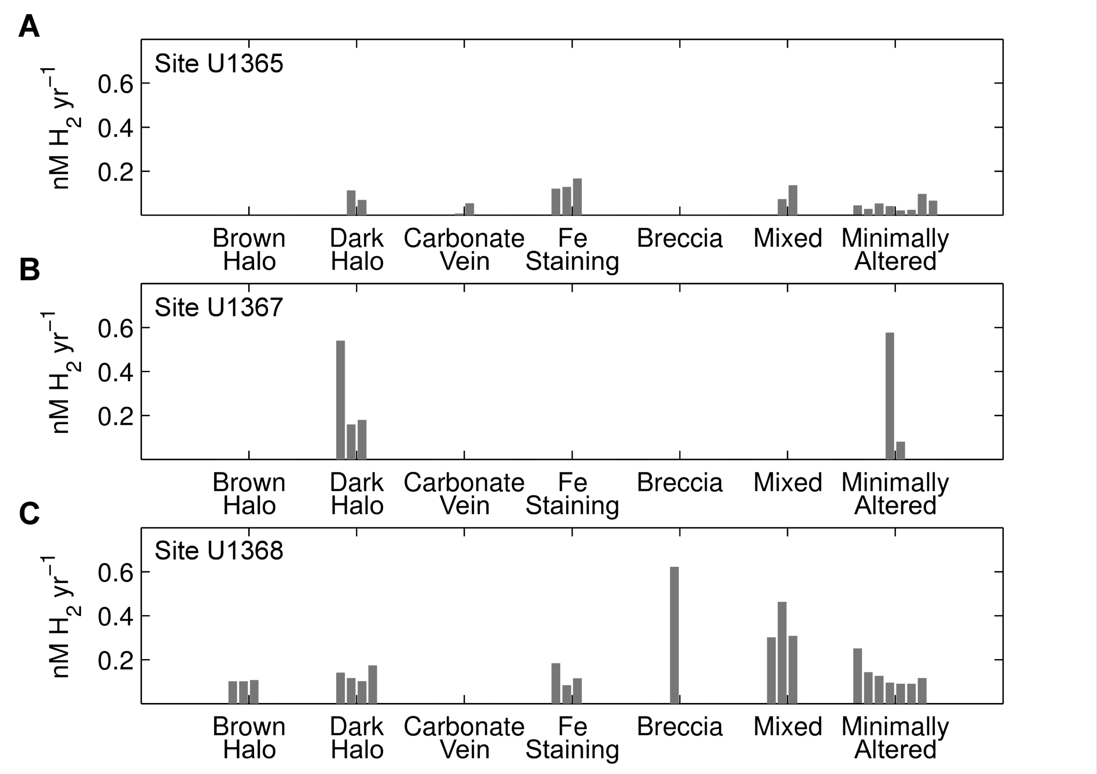

Figure S1. Comparison of volume-normalized radiolytic H_2 production rates for the different alteration types. The rates shown here are for 1 μm fractures at Sites (A) U1365, (B) U1367, and (C) U1368.
